# Hydroxyapatite particles substituted with Pd ions for remarkable antibacterial performance

**DOI:** 10.3389/fchem.2025.1698673

**Published:** 2025-09-25

**Authors:** Seung-Jae Jeong, Yoon-Seop Jeong, Jae-Won Jeong, Heesoo Lee, Young-Tae Kwon

**Affiliations:** 1 New-Functional Powder Materials Research Center, Korea Institute of Materials Science, Changwon, Republic of Korea; 2 School of Materials Science and Engineering, Pusan National University, Busan, Republic of Korea

**Keywords:** hydroxyapatite particles, Pd substitution, antibacterial performance, aerosol reaction, ultrasonic spray pyrolysis process

## Abstract

The increasing threat of bacterial infections to human health has positioned the development of antibacterial materials as a critical global research priority. Recently, hydroxyapatite (HAP), which is chemically similar to the main components of bone and teeth, has attracted considerable attention as a promising antibacterial material due to its ability to inhibit bacterial adhesion and proliferation through electrostatic repulsion. However, hydroxyapatite exhibits lower antibacterial activity compared to metal particles or metal ions, which remains a limitation for its application as an antibacterial agent. Here, we present simple and one-step synthesis of the hydroxyapatite particles partially substituted with palladium (Pd) ions. The designed reaction simultaneously allows the formation of HAP particles and the substitution of Calcium ions (Ca^2+^) with Pd^2+^ ions within the HAP lattice. While the pure HAP particles show an antibacterial activity of approximately 97.5%, Pd-5% substituted HAP demonstrates ultrahigh antibacterial performance exceeding 99.9% against three different bacteria, including *Staphylococcus aureus*, *Klebsiella pneumoniae*, and *Escherichia coli*. This study comprehensively investigates the correlation between the Pd substitution and antibacterial ability, providing valuable insights for the development of advanced antibacterial materials aimed at promoting human health and a safe, clean environment.

## 1 Introduction

Bacterial infections, which are recognized as a major global challenge to human health, were the second leading cause of death in 2019 (Allegranzi et al., 2011; Rosenthal, 2011; Rutledge-Taylor et al., 2012; Zhang C. et al., 2024). To mitigate the threat of bacterial infections, the development of antibacterial surfaces and materials has attracted considerable attention across various aspects of daily life, including kitchenware, schools, and healthcare facilities (Levy and Marshall, 2004; Schäberle and Hack, 2014). Controlling surface charge or wettability is a simple and effective way to prevent bacterial adhesion which is primarily driven by Coulombic, van der Waals, and hydrogen bonding interactions (Li et al., 2021). However, the surface modification for preventing bacterial adhesion have inherent limitations, including non-bactericidal nature, susceptibility to environmental conditions and surface fouling, and difficulty in universally preventing adhesion of diverse bacterial species (Dou et al., 2015; Zhao et al., 2021; Antifouling and ant ibacterial properties). An alternative approach to antibacterial effect is to induce bacterial cell elimination. Antibacterial materials capable of altering or disrupting cellular components are considered an effective strategy due to their broad-spectrum antibacterial activity and lower potential for inducing bacterial resistance (Li et al., 2021).

The inorganic metal particles with high ion exchange and sorptive capacities are representative antibacterial materials (Gao et al., 2009; Hegab et al., 2016; Tang et al., 2017; Wahid et al., 2017; Yang et al., 2006). The metal ions (e.g., Ag^+^, Cu^2+^, and Fe^2+^) released from the metal particles can interact with bacterial membranes and enter cells through ion channels. The penetrated metal ions in the bacteria cell catalyze Fenton reaction to produce excessive reactive oxygen species (ROS). These ROS damage the cell membrane and cause oxidative stress, ultimately leading to bacterial inactivation (Lam et al., 2022; Ning et al., 2015; Saidin et al., 2021; Zhang B. et al., 2024; Zhao et al., 2022). Despite their excellent performance, antibacterial metal particles suffer from critical limitations of easy aggregation and oxidation in practical applications. In particular, the oxidation of metal particles not only exhibits reduction in antibacterial activity but also causes toxic effects to human health. Another promising material is hydroxyapatite (HAP) particles which constitute approximately 60%–70% of the inorganic component of the bone matrix. HAP particles exhibit a high ion-exchange capacity for various cations, contributing to their excellent biocompatibility and bioactivity (Ragab et al., 2014). Furthermore, the low cost and high oxidative stability of HAP offer enhanced reliability for use as an antibacterial material in various industrial applications. However, the relatively low antibacterial performance of HAP compared to metallic particles remains a challenge, prompting continued research efforts to enhance its antibacterial efficacy.

This work introduces a strategy to enhance the relatively low antibacterial performance of HAP by partially substituting Ca^2+^ sites with trace amounts of palladium (Pd) ions. The excellent catalytic activity of Pd facilitates the generation of ROS, including hydrogen peroxide (H_2_O_2_), superoxide anions (O_2_^−^), and hydroxyl radicals (·OH), which contribute to their antibacterial activity. The integration of aerosol processing with chemical reactions allows a one-step synthesis of hydroxyapatite particles partially substituted with Pd ions (referred to as “Pd-HAP”), thereby contributing to the development of advanced antibacterial materials. We demonstrate that the incorporation of a small amount of Pd into the HAP lattice enables ultrahigh antibacterial performance, achieving over 99.9% reduction against three different bacterial strains: *S. aureus* (*Staphylococcus aureus*), *K. pneumoniae* (*Klebsiella pneumoniae*), and *E. coli* (*E. coli*).

## 2 Experimental

### 2.1 Materials

Calcium nitrate tetrahydrate (Ca(NO_3_)_2_·_4_H_2_O, ACS reagent, 99%) and Palladium (II) chloride (PdCl_2_, 99%) were purchased from Sigma-Aldrich. Phosphoric acid (H_3_PO_4_, Extra Pure, 99.9%) were purchased from DC Chemical Co., Ltd.

### 2.2 Manufacturing for HAP and Pd-HAP particles

To synthesize HAP particles, calcium nitrate tetrahydrate (1.0 M) and Phosphoric Acid (0.6 M) were dissolved in deionized (DI) water. The solution for Pd-HAP particles was prepared by mixing calcium nitrate tetrahydrate (0.95 M), phosphoric acid (0.6 M), and palladium chloride (0.05 M) in DI water. Each prepared solution was transferred into a 100 mL syringe and delivered into a quartz glass tube at a constant flow rate of 0.4 mL/min. Subsequently, the solutions were atomized using an ultrasonic nebulizer and introduced into the ultrasonic-assisted spray pyrolysis (USP) system. The ultrasonic nebulizer produced aerosol droplets, which were transported through the system by a dry air stream. The gas was used at a flow rate of 5 L/min. Two distinct thermal zones were established in the reaction chamber. The first heating zone was maintained at 200 °C. To investigate the temperature-dependent structural characteristics of the synthesized particles, the second heating zone was set to 600, 700, and 800 °C. The manufactured HAP and Pd-HAP particles were collected in the collecting zone using a filter paper.

### 2.3 Characterization

The structural morphology of as-synthesized samples was characterized via field-emission scanning electron microscopy (FE-SEM; JSM-7610F, JEOL). The crystal structure was confirmed via X-ray diffraction (XRD; Rigaku D/Max-2500VL/PC). High-performance X-ray Photoelectron Spectrometer (XPS; VG Scientific ESCALAB 250) was utilized to identify the substitution of Pd ion within the HAP particles. Brunauer–Emmett–Teller (BET; TriStar II 3020, Micrometrics) analysis was conducted to determine the specific surface area and pore size of the synthesized particles. To confirm the antibacterial activity of manufactured particles, tests used three different cells, including *S. aureus*, *K. pneumoniae*, and *E. coli*. Each bacteria cell was cultured in two different conditions-control; HAP or Pd-HAP particles—in an incubator at 37 °C with 5% CO_2_. The bacterial reduction rate was measured by counting the bacterial concentration after 24 h.

## 3 Results and discussion

**FIGURE 1:**
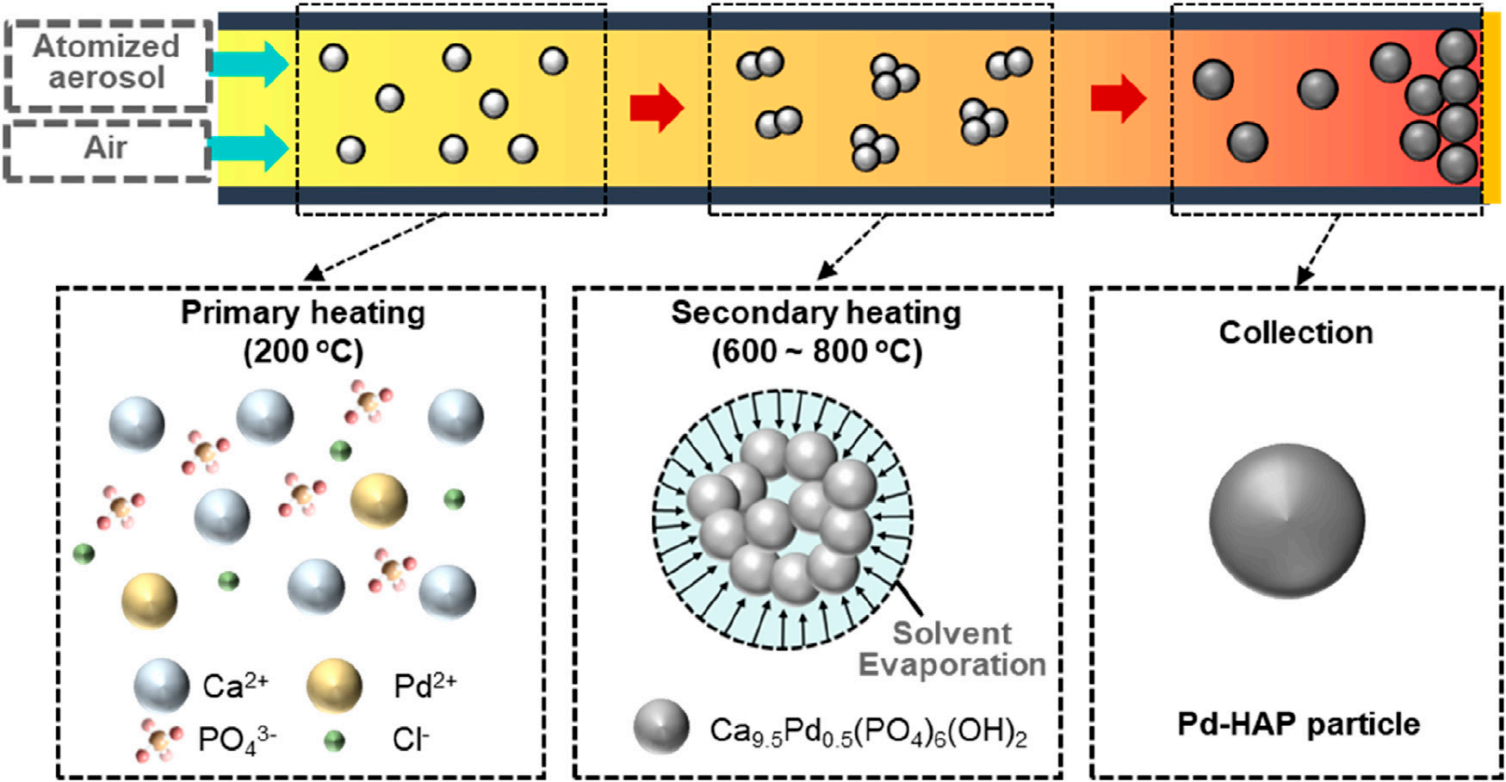
Schematic illustration of the manufacturing strategy for the hydroxyapatite (Pd-HAP) particles partially substituted with Pd ions. The HAP particles are fabricated using the ultrasonic-assisted spray pyrolysis (USP) method which includes two thermal zones of primary and secondary heating. The aerosol reaction between calcium nitrate and phosphate ion results in the formation of spherical HAP particles.

Figure 1 shows the manufacturing strategy for the spherical HAP particles partially substituted with Pd ions. USP process involves a series of steps, including precursor atomization, solution pyrolysis, and subsequent solid formation, resulting in the production of micro- and nano-sized particles (Majerič and Rudolf, 2020; Rahemi Ardekani et al., 2019; Se Chang et al., 2023). Ultrasonic nebulization of aqueous metal precursor solutions generates fine aerosol droplets, which are subsequently transported through a dual-zone high-temperature reactor. Within this thermal environment, the droplets undergo rapid solvent evaporation, shrinkage, solute precipitation, and particle sintering, while heat-induced chemical reactions simultaneously occur inside the droplets (Workie et al., 2023). We investigated the temperature-dependent structural characteristics of the synthesized particles by adjusting the second heating zone of the USP process to 600, 700, and 800 °C. In addition, two types of particles—pure HAP and Pd-substituted HAP (Pd-HAP)—were synthesized to investigate the effect of Pd ion substitution within the HAP structure. In terms of the HAP particles, Ca nitrate and phosphoric acid were initially dissolved in DI water. At the primary heating stage of 200 °C, the aqueous solution starts to evaporate, initiating nucleation and leading to the formation of Ca_10_(PO_4_)_6_(OH)_2_. During the secondary heating above 600 °C, the HAP nuclei undergo growth followed by subsequent sintering. The sintering process reduces the surface energy and promotes the formation of spherical HAP particles. The chemical reaction can be indicated as follows:

$$\text{Ca}{\left({\text{NO}}_{3}\right)}_{2}\cdot 4H_{2}O\;\to \text{ Ca}{\left({\text{OH}}_{2}\right)}_{6}^{2+}\;\left(\text{aq}\right)+2{\text{NO}}_{3}^{‐}\;\left(\text{aq}\right)$$

$$H_{3}{\text{PO}}_{4}\;\to \;3H^{+}\;\left(\text{aq}\right)+{\text{PO}}_{4}^{3‐}\;\left(\text{aq}\right)$$

$$10\text{Ca}{\left({\text{OH}}_{2}\right)}_{6}^{2+}\;\left(\text{aq}\right)+6{\text{PO}}_{4}^{3‐}\;\left(\text{aq}\right)\to {\text{Ca}}_{10}{\left({\text{PO}}_{4}\right)}_{6}{\left(\text{OH}\right)}_{2}\;\left(s\right)+18H_{2}O\;\left(g\right)$$

For Pd-HAP particles, Pd source corresponding to 5% of the Ca concentration was added to the aqueous solution containing Ca and P. The aerosol chemical reaction can be described as follows:

$${\text{PdCl}}_{2}\;\to \;{\text{Pd}}^{2+}\;\left(\text{aq}\right)+2{\text{Cl}}^{‐}\;\left(\text{aq}\right)$$

$$9.5\text{Ca}{\left({\text{OH}}_{2}\right)}_{6}^{2+}\;\left(\text{aq}\right)+6{\text{PO}}_{4}^{3‐}\;\left(\text{aq}\right)+0.5{\text{Pd}}^{2+}\;\left(\text{aq}\right)\;\to \;{\text{Ca}}_{9.5}{\text{Pd}}_{0.5}{\left({\text{PO}}_{4}\right)}_{6}{\left(\text{OH}\right)}_{2}\;\left(s\right)+18H_{2}O\;\left(g\right)$$

**FIGURE 2:**
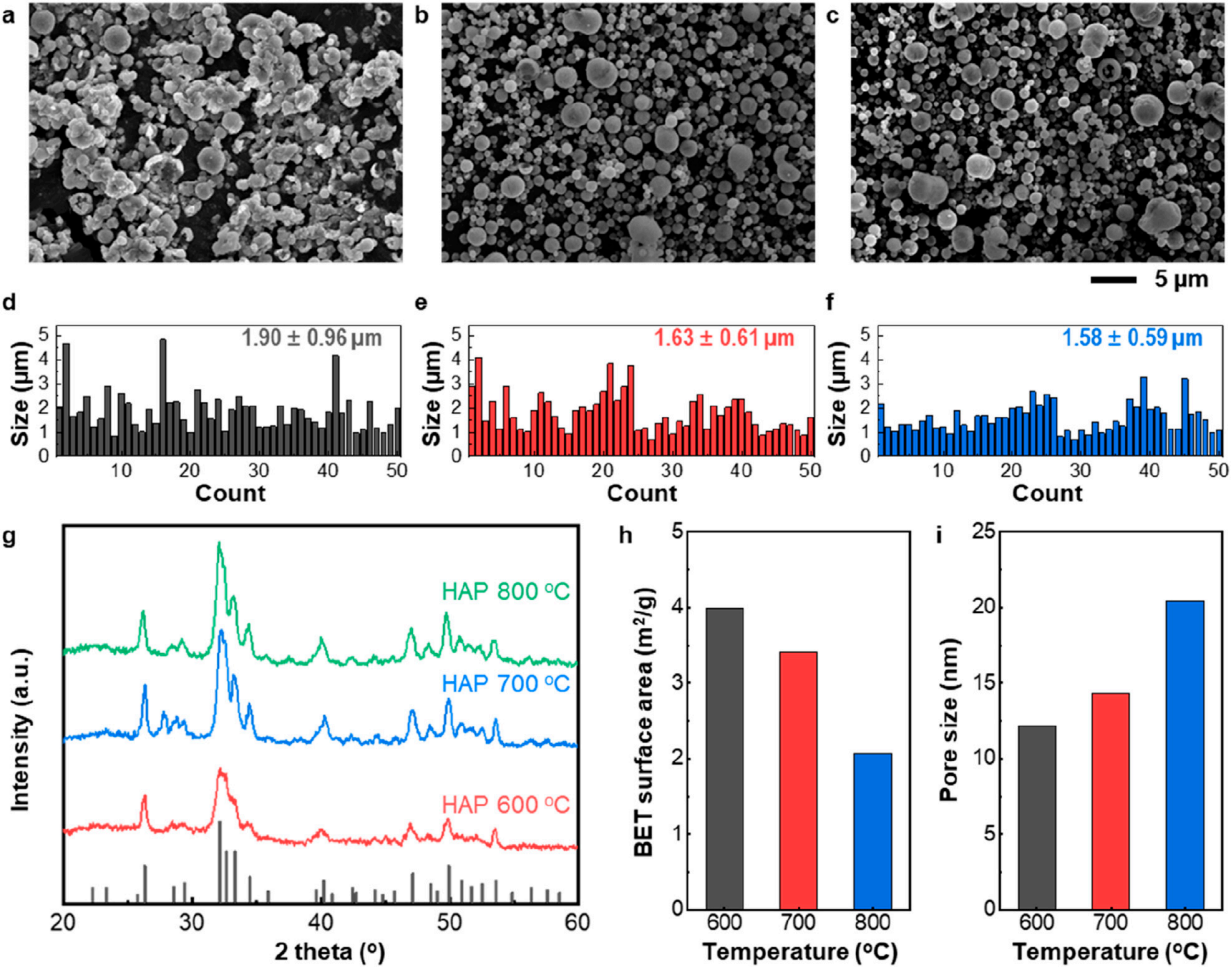
Structural characterization of the as-synthesized HAP particles. **(a–c)** Scanning electron microscopy (SEM) images of HAP particles manufactured via the USP process at **(a)** 600 °C, **(b)** 700 °C, and **(c)** 800 °C. **(d–f)** Particle size distributions corresponding to the HAP samples synthesized at **(d)** 600 °C, **(e)** 700 °C, and **(f)** 800 °C. **(g)** X-ray diffraction (XRD) patterns of HAP particles obtained at 600 °C, 700 °C, and 800 °C, respectively. The crystal structure of as-synthesized HAP particles is identified based on JCPDS card No.00-009-0432. **(h)** Specific surface area and **(i)** pore size analysis of HAP particles prepared at different secondary heating temperatures.

Figures 2a–c presents the morphology and structural features of HAP synthesized in the secondary heating zone of the USP process at temperatures ranging from 600 °C to 800 °C, as revealed by electron microscopy images. The HAP synthesized at 600 °C exhibits irregular morphology (Figure 2a), whereas the samples prepared at 700 °C and 800 °C display well-defined spherical structures (Figures 2b,c). The size distribution analysis further indicates that the 600 °C sample (Figure 2d) has a broader size distribution and a larger average diameter compared to the particles synthesized at higher temperatures (Figures 2e,f). These results suggest that elevated temperatures enhance particle sintering, leading to the formation of more uniform spherical morphologies. As shown in Figure 2g, the XRD patterns of all HAP particles exhibit strong diffraction peaks at 26.8°, 32.5°, 33.1°, and 34.6°, which are consistent with the standard hydroxyapatite phase (JCPDS No. 00-009-0432). Notably, sharper peaks are observed in the samples prepared at higher temperatures, indicating improved crystallinity, which is consistent with the SEM findings. Figure 2h and i show the specific surface area and pore size of the HAP particles. As the synthesis temperature increases, the aerosol reaction induces accelerated sintering, resulting in a decrease in specific surface area and the formation of larger pores.

**FIGURE 3:**
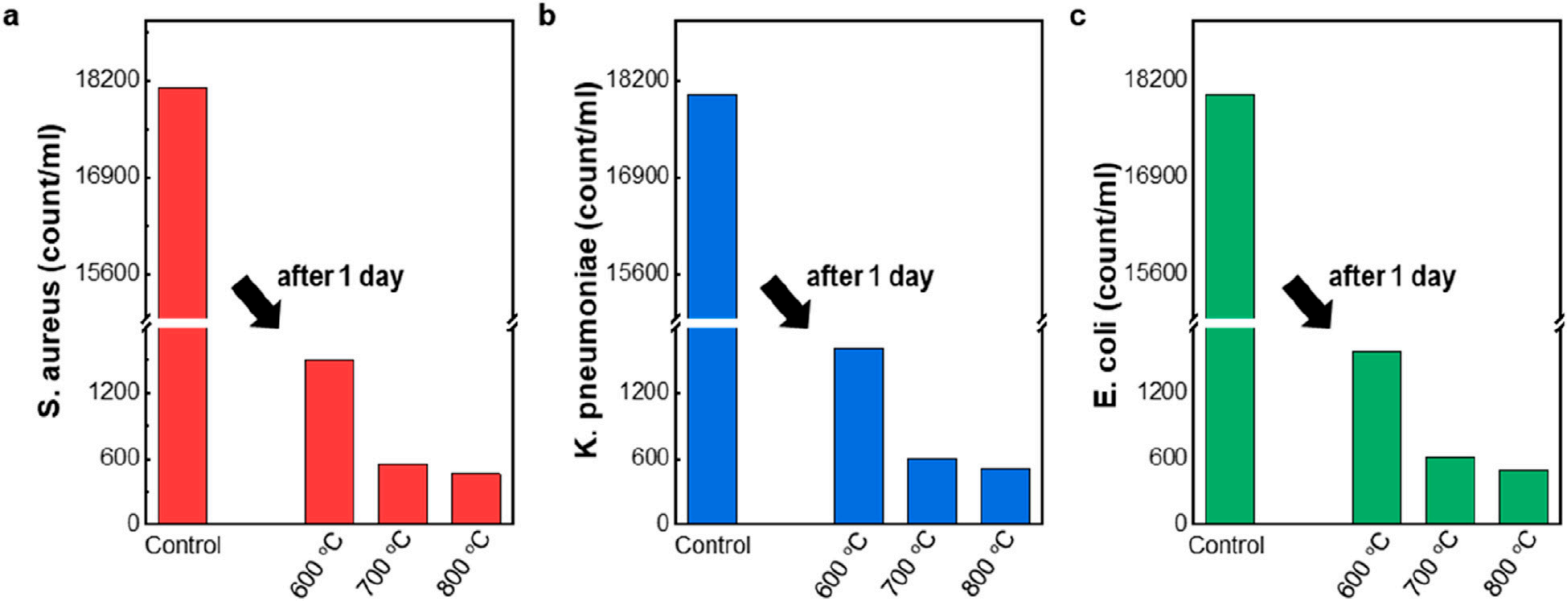
Antibacterial activity of the manufactured HAP particles. **(a–c)** Antibacterial activity against **(a)** *Staphylococcus aureus* (*S. aureus*), **(b)** *Klebsiella* pneumonia (K. pneumonia), and **(c)** *Escherichia coli* (*E. coli*), represented by the colony number in the control experiment to the colony numbers in antibacterial experiments.

Figure 3 displays the antibacterial activity of HAP particles against *S. aureus*, *K. pneumoniae*, and *E. coli*. The bacteria *S. aureus*, *K. pneumoniae*, and *E. coli* are widely used as representative strains because of their clinical importance and distinct cell wall compositions. Testing against these species allows for assessment of antibacterial activity against both Gram-positive (*S. aureus*) and Gram-negative (*K. pneumoniae* and *E. coli*) bacteria (Mah and O'Toole, 2001; Wiegand et al., 2008). We evaluated the antibacterial performance of pure HAP particles synthesized at secondary heating zone temperatures of 600, 700, and 800 °C. The HAP particles synthesized at 600, 700, and 800 °C exhibit antibacterial activities of 91.3%, 96.6%, and 97.4% against *S. aureus*, respectively (Figure 3a). Against *K. pneumoniae* (Figure 3b), the corresponding killing efficiencies are 91.1%, 96.6%, and 97.1%. Similarly, the samples tested against *E. coli* (Figure 3c) show antibacterial activities of 90.8%, 96.5%, and 97.2%, respectively. All HAP samples exhibit antibacterial activity compared to the control group; however, those synthesized at higher temperatures consistently show enhanced performance against all tested bacteria. Although the HAP particles synthesized at 600 °C possess a larger specific surface area, the enhanced crystallinity of the 800 °C sample appears to play a more critical role in achieving superior antibacterial activity.

**FIGURE 4:**
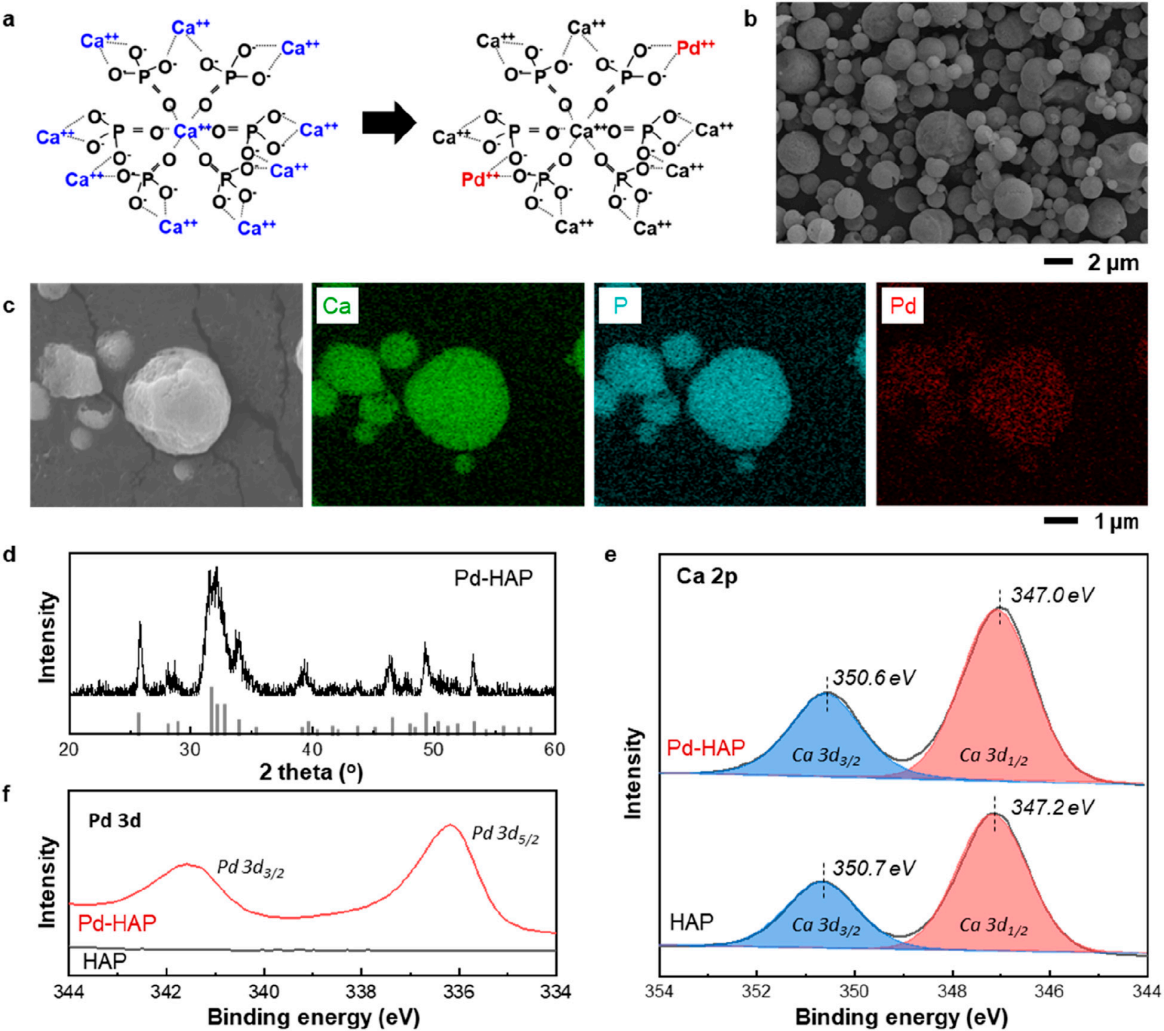
Structural characterization of HAP particles partially substituted with Palladium (Pd). **(a)** Illustration showing the partial substitution mechanism of Pd within the HAP molecular structure. **(b)** SEM image of HAP particles partially substituted with Pd (referred to as Pd-HAP). **(c)** High-resolution SEM image and the corresponding elemental mapping of Ca, P, and Pd in Pd-HAP samples. **(d)** XRD result indicating the structure of as-synthesized Pd-HAP. **(e)** Ca 2p and **(f)** Pd 3d X-ray photoelectron spectroscopy (XPS) spectra of both HAP and Pd-HAP samples.

**FIGURE 5:**
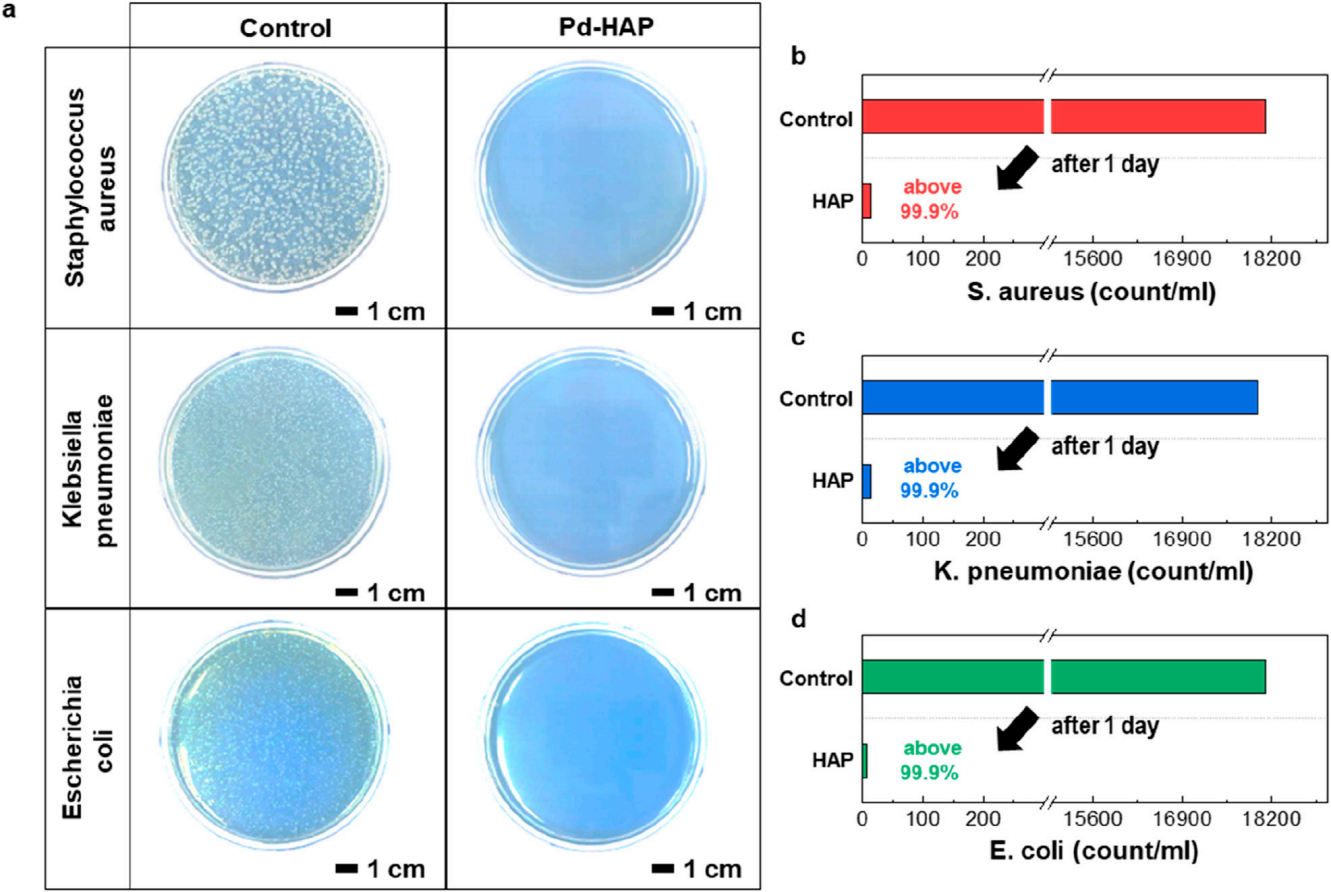
Antibacterial activity for the Pd-HAP particles. **(a)** Photographs of colony-forming cell assays against *S. aureus*, K. pneumonia, and *E. coli*. Left images present the original cell array (control sample), whereas right photos show the cell array exposed to Pd-HAP. **(b–d)** Quantitative comparison of colony formation in control and antibacterial conditions for **(b)** *S. aureus*, **(c)** *K. pneumoniae*, and **(d)** *E. coli*.

To maximize the antibacterial performance of HAP particles, we synthesized a Pd-substituted HAP by partially replacing Ca^2+^ ions with a small amount of Pd^2+^. The molecular structures of HAP illustrated in Figure 4a depict the strategy for Pd substitution. Considering its high crystallinity and strong antibacterial reactivity, the Pd- HAP was synthesized at 800 °C. The as-synthesized Pd-HAP particles show exhibit similar size and distribution to those of the pure HAP particles (Figure 4b). Figure 4c indicates the high-resolution SEM image and the corresponding elemental mapping of Ca, P, and Pd. The uniform distribution of Pd within the interior of the HAP particles is confirmed, while maintaining their original morphology. The XRD pattern shown in Figure 4d demonstrates preservation of the pure HAP crystal structure without the formation of any secondary phases associated with Pd. XPS is used to analyze the composition and chemical states of manufactured particle (Figures 4e,f). In Figure 4e, the high-resolution XPS Ca 2p could be curve-fitted to Ca 2p3/2, Ca 2p1/2 of the Ca(II) state doublet peaks (350.7 and 347.2 eV) (Lu et al., 2000; Maachou et al., 2013). After substitution reaction, Ca 2p peaks slightly shift toward lower binding energy, indicating that the Pd ion substitution alters the electronic structure and chemical environment surrounding the Ca sites. Additionally, the emergence of distinct Pd 3d binding energy peaks confirms the successful incorporation of Pd ions into the HAP lattice through substitution (Figure 4f). To demonstrate the enhanced antibacterial effect of Pd substitution, we conducted a colony-forming unit assay, as presented in Figure 5. The control groups against each bacteria were also investigated for comparison. After incubation with the Pd-HAP particles, Figure 5a shows that all visible white colonies corresponding bacteria are eliminated in comparison to the control group. Furthermore, the culture medium treated with Pd-HAP became transparent, indicating effective sterilization of bacteria cells. In a quantitative comparison graph between the control and Pd-HAP, all bacteria cells, including *S. aureus* (Figure 5b), *K. pneumoniae* (Figure 5c), and *E. coli* (Figure 5d), are removed by more than 99.9%. Taken together, the findings confirm that even a small amount of Pd substitution leads to an ultrahigh antibacterial performance compared to the pure HAP.

## 4 Conclusion

This paper reports a simple yet effective Pd substitution strategy to achieve remarkable antibacterial performance of non-metallic HAP materials. The aerosol chemical reaction in the USP process enables the one-step synthesis of Pd-substituted HAP particles by simultaneously forming HAP and incorporating Pd ions. By controlling the secondary heating temperature, we optimized the crystallinity and morphology of the HAP particles, exhibiting well-defined spherical shapes and superior antibacterial activity. Furthermore, the partial substitution of Ca^2+^ with a small amount of Pd^2+^ ions significantly boosted the antibacterial efficacy of HAP particles. Overall, this work presents a practical strategy for enhancing the antibacterial performance of HAP through controlled crystallization and Pd substitution. The findings suggest that Pd-HAP holds strong potential for applications in biomedical and environmental fields where effective antibacterial materials are critically required.

## Data Availability

The original contributions presented in the study are included in the article/supplementary material, further inquiries can be directed to the corresponding authors.
